# The effect of white coats and gender on medical students’ perceptions of physicians

**DOI:** 10.1186/s12909-017-0932-1

**Published:** 2017-05-26

**Authors:** Malika Ladha, Aleem Bharwani, Kevin McLaughlin, Henry T. Stelfox, Adam Bass

**Affiliations:** 1 Department of Medicine, University of Calgary, 1403 29th Street NW, Calgary, AB T2N 2T9 Canada; 2 Departments of Critical Care, Medicine and Community Health Sciences, University of Calgary, Calgary, Canada

**Keywords:** Attire, Bias, Gender

## Abstract

**Background:**

Despite the fact that medical schools spend a considerable effort to rate clinical instructors, there is limited evidence regarding the effect of physical characteristics on instructor ratings. White coats have been shown to alter patients’ perceptions of physicians although it has not been determined if preceptors who wear white coats are rated differently than their colleagues.

**Methods:**

Second year medical students were administered a questionnaire with four clinical scenarios depicting medical errors accompanied by a picture of a physician of different sexes and ethnicities. The packages were randomized so that the physicians depicted either had or did not have a white coat.

**Results:**

White coats did not alter the perception of physicians’ ratings by medical students although sex and ethnicity/case were associated with the perception of trustworthiness, physician management, competence, professionalism and the perception of medical error.

**Conclusions:**

Physical characteristics may alter students’ ratings of physicians.

**Electronic supplementary material:**

The online version of this article (doi:10.1186/s12909-017-0932-1) contains supplementary material, which is available to authorized users.

## Background

Medicine is a discipline that is taught through apprenticeship. Medical students are endowed with basic medical knowledge throughout medical school but it is the interaction with physicians in a clinical context that ultimately crystallizes this information. For this reason, medical schools make a considerable effort in trying to evaluate and determine the effectiveness of teachers. As such, a considerable amount of research has been concentrated on trying to define which characteristics make a competent and effective teacher [1–4]. For the most part, research indicates that teaching effectiveness is a combination of both non-cognitive and cognitive skills. Some particular characteristics that have been shown to define effective teachers include: good clinical knowledge, communication skills, concern for learners, commitment to teaching, and communication [1–4]. However, the surveys used to determine the characteristics of teaching effectiveness have not concentrated on other features, such as physical attributes, that could affect perceived teaching ability or competence.

It is a well-known phenomenon in patient centered literature that physical characteristics affect perceived competence of physicians [5]. A recent meta-analysis of studies on physician attire on patients’ perceptions demonstrated that a preference or positive relationship of formal attire was found in the majority of studies [5]. Patients who have a better relationship with their physician are more likely to adhere to treatment plans and disclose information [5–7]. Given that medical competence is a defining characteristic of a good medical educator, it is possible that physical attributes play a role in the perception of clinical competence. Recently, Rannelli et al. found that physical attractiveness was a modifier of so-called “charisma” and that being more attractive had a positive correlation with teacher evaluation ratings [8]. Given that medical students usually have no pre-existing relationships with physician-preceptors and that their encounters may occur over short time frames, it is possible that students may unconsciously establish clinical competence based on physical appearance. However, the effect of physicians’ physical characteristics on perceived competence has not been well studied in the domain of medical education.

We aimed to determine if particular components of the physical appearance affected students’ ratings of competence. In particular, given that white coats are modifiable physical attribute and are frequently seen as an eminent symbol of the profession’s status and competence, [9] we examined the role that white coats had on students’ perception on dimensions of competence and judgement making abilities. In this study, we asked second year medical students to rate the competencies and abilities of physicians – either pictured with or without a white coat over professional clothing – based on written clinical scenarios. The aim of our study was to explore whether white coats impact learner perceptions of physicians in these situations. We hypothesized that medical students would be positively biased towards those who wore white coats over those who opted for professional attire without white coats.

## Methods

### Study design

This cross-sectional study used a self-administered questionnaire. Ethics approval was obtained from the University of Calgary Conjoint Research Ethics Board.

### Participants

The University of Calgary medical school operates a three-year undergraduate curriculum: the first two years consists of system-based courses, and the final year is a clinical clerkship. The class of 2016 is 155 students and includes 58 males, 97 females. One hundred and twenty four medical second year medical students from the class of 2016 participated in this study during a break in between lectures. These participants had just completed pre-clerkship clinical experiences, thus providing them with a context to judge medical errors. Each participant underwent an informed consent process prior to completing the questionnaire.

### Materials/procedure

We created a questionnaire with four clinical scenarios portraying potential physician errors (see Additional file 1). The errors were created to introduce some uncertainty in a physician’s performance in order avoid a ceiling effect on ratings of physicians. Each hypothetical scenario was paired with a picture of an actor depicting a physician who would have had no previous interaction with participants. The actor was either pictured with or without a white coat. The identical cases were given to both groups with the only difference in each case being that physicians were wearing or not wearing a white coat. While the gender and race of each case was different, each comparator was always white coat versus non-white coat. Actors represented both male/female sexes and various ethnicities. The participants in this study were under the impression that the actors were real life family physicians.

Survey packages were randomly distributed such that half of the participants received questionnaires with photographs of actors wearing white coats and the other half of participants received photographs of actors without white coats. Block randomization in groups of ten surveys was used to ensure balanced groups. Participants were unaware of the bias being tested; they were misdirected towards the true intent of the study.

After reading each stem, participants were asked to rate a number of domains regarding the physicians’ qualities (competence, trustworthiness, professionalism), appropriateness of actions, and whether the physician had committed an error using a 5-point Likert scale. Upon completion of the study, the true intent of the research was revealed to the students.

### Statistical analysis

We used multiple linear regression where our outcome variables were ratings of appropriateness of actions, physician competence, physician trustworthiness, physician professionalism and the perception of whether or not a medical error was committed. Any portion of a questionnaire that was completed was included in our analysis, and uncompleted portions were not included in our analysis. Our explanatory variables were the presence of a white coat versus no white coat, sex (male versus female) and case/caucasion race versus non-caucasion race. Case and race could not be evaluated independently since we had a different ethnicity for each case. We considered interaction between explanatory variables in our regression model. If we found a significant interaction, we reported the stratified analyses. All data was analyzed using STATA version 11.0 (StataCorp LP, College Station, TX, USA).

## Results

One hundred and twenty four out of one hundred and fifty five medical students offered to participate completed the questionnaire (*n* = 124). One questionnaire in each of the randomization groups was not completed in its entirety. The portions of incomplete questionnaires that had been filled out were also included in the analysis. The aggregated data is listed in Table 1.

**Table 1:** Ratings of white coat (WC) versus non-white coat (NWC) for each of the four clinical scenarios using a Likert scale from 1 to 5 (higher rating is more agreement with the statement)

	Scenario 1 (Female)		Scenario 2 (Male)		Scenario 3 (Female)		Scenario 4 (Male)		Aggregate	
	WC	NWC	WC	NWC	WC	NWC	WC	NWC	WC	NWC
Appropriateness of Actions	1.94	1.92	3.45	3.23	1.54	1.61	3.71	3.69	3.48	3.51
Competence	2.90	3.21	3.67	3.66	2.34	2.49	3.31	3.41	2.66	2.61
Trustworthiness	2.74	3.03	3.50	3.57	2.17	2.09	3.68	3.72	3.05	3.20
Professionalism	2.46	2.60	3.44	3.4	2.19	2.18	3.82	3.92	3.02	3.11
Was an error committed?	3.61	3.74	2.47	2.61	4.34	4.15	3.52	3.53	2.98	3.02

In our model, we found no differences when examining the association of white coat on ratings of appropriateness of actions, physician competence, physician trustworthiness, and physician professionalism between the groups. There was no difference of white coats altering the perception of whether or not a medical error was committed.

In our study, differences were observed when we examined the effect that sex or case/race had on ratings of qualities of appropriateness of actions, physician competence, physician trustworthiness, and physician professionalism. For physician trustworthiness male sex (regression coefficient [β] = 1.11, 95% confidence interval [CI] 0.96–1.27; *p* < 0.001) was found to have a positive correlation (R^2^ of 0.28). For the remaining domains, both sex and case/Caucasian race were significant. For appropriateness of actions, male sex (β = 1.96, 95% CI 1.77–2.14; *p* < 0.001) was positively correlated and case/Caucasian race (β = −0.38, 95% CI -0.59– -0.16; *p* < 0.001) was negatively correlated (R^2^ of 0.5281). For physician competence, male sex (β = 0.63, 95% CI 0.44–0.83; *p* < 0.001) and case/Caucasian race (β = 0.29, 95% CI 0.058–0.52; *p* < 0.014) were both positively correlated (R^2^ of 0.1647). For physician professionalism male sex (β = 1.52, 95% CI 1.35–1.69; *p* < 0.001) was positive correlated and case/Caucasian race (β = −0.47, 95% CI -0.67– -0.27; *p* < 0.001) was negatively correlated (R^2^ 0.4174). In the area of medical error, male sex (β = −0.44, 95% CI -0.65– -0.22; *p* < 0.001) and case/Caucasian race (β = −0.98, 95% CI -1.22– -0.73; *p* < 0.001) were negatively correlated with a perception of error (R^2^ 0.2575). As such, male gender and case/Caucasian race were viewed as protective from being ascribed error. These results are summarized in Table 2.

**Table 2:** Beta coefficients and R2 of the different questionnaire questions

	White Coat (β)	Male Sex (β)	Case/Caucasian Race (β)	*R* ^2^
Appropriateness of Actions	0.04 (−0.12–0.20) *p* = 0.6, *t* = 0.53	1.96 (1.77–2.14) *p* = <0.001, *t* = 20.8	−0.38 (−0.59 – −0.16) *p* = 0.001, *t* = −3.47	0.5281
Competence	−0.14 (−0.31–0.03) *p* = 0.09, *t* = −1.7	0.63 (0.44–0.83) *p* = < 0.001, *t* = 6.32	0.29 (0.058–0.52) *p* = 0.01, *t* = 2.47	0.1647
Trustworthiness	−0.08 (−0.24–0.08) *p* = 0.3, *t* = −1.01	1.11 (0.96–1.27) *p* = <0.001, *t* = 13.76		0.28
Professionalism	−0.05 (−0.19–0.10) *p* = 0.5, *t* = −0.69	1.52 (1.35–1.69) *p* = <0.001, *t* = 17.4	−0.47 (−0.67 – −0.27) *p* = <0.001, *t* = −4.63	0.4174
Was an error committed?	−0.03 (−0.21–0.15) *p* = 0.7, *t* = −0.37	−0.44 (−0.65 – −0.22) *p* = <0.001, *t* = −4.02	−0.98 (−1.22 – −0.73) *p* = <0.001, *t* = −7.74	0.2575

The beta, *p* and *t* values of white coat are added for all regressions while only the significant beta, p and t values are shown for other the other comparators

## Discussion

Medical schools spend a considerable amount of effort and expense to ensure that physicians are rated regularly to monitor the quality of instruction. Additionally, these ratings are used as sources of promotion or as sources to determine educational awards.

While our initial hypothesis that white coats would alter students’ perceptions of physicians was disproven, our study demonstrated that gender and possibly race altered students’ ratings of the physicians in simulated clinical scenarios. The strength of the relationship between these attributes was variable but explained up to 52% of the variance in the context of trustworthiness. Notably, the perception of medical error in our cases was correlated with gender and case/Caucausian race, albeit weak.

As suggested by Rannelli et al., our study adds to the literature by suggesting that superficial physical traits may have an impact on physicians’ ratings by medical students [8]. While students may not be conscious that they are influenced by physical characteristics, these observations seem consistent with the theory of impression formation, a dual processing model used to rate other individuals [10, 11]. This theory suggests that impressions start with stereotypes, which inform the rater’s initial a priori expectations of the situation. However, raters alter their appraisal of an individual as they gain insight into their behaviour. This dual model is based on the presence of both implicit and explicit memory [12, 13]. Implicit memory enables storage of previous experiences which inform our average expectations of any given context. In contrast, explicit memory provides rules which we apply when rating others’ behaviours. In our situation, the effect of gender and case/race persisted despite clinical information suggesting that these biases may be strong. While it is impossible for medical schools to eliminate bias in the evaluations process, it is possible that medical schools could monitor for bias in evaluations and act accordingly. At the very least, schools should be aware that bias could be having an impact on the evaluation of their faculty.

The presence of a white coat did not portend a better rating in our study. Even if an effect existed at a superficial level, it is appears to have been washed away by the context of the clinical information provided to the students. As to why white coats had a minimal impact on the results, it is possible that learners’ pre-exposure to white coats may have resulted in a decreased association of this symbol as a marker of competency and professional behaviour. Additionally, physicians’ behaviour or dressing habits in the hospital could have resulted in a diminished symbolism of the white coat in our center, or alternatively students now feel incorporated into this group.

Our study has limitations that may impact the interpretation of our results. The analyses in this paper are exploratory and should be interpreted with caution. While we tried to create realistic clinical scenarios, it is possible that students’ impressions would have been different in a real-life context after talking or interacting with a physician. Unfortunately, we did not collect the gender or race of students that completed the survey and therefore we are unable to tease out the effect that these had on the outcomes. However, based on class demographics we can be confident that there was a majority of women who completed the study due to the fact that even if all the men in the class had participated (which is unlikely) they still would have formed a minority of participants. It could be argued that we did not show students the pictures without clinical information to determine a baseline rating to allow further interpretation of the results. We feel this condition was completely unrealistic and only biases that endure despite clinical information would have any real world implications. Finally, while we tried to standardize the seriousness of errors across the groups, however, there may have been variability in how the students would have viewed the seriousness of these errors. It should also be noted that this study was completed in a single centre which limits the generalizability of our results. Finally, only second year medical students were included and while it is unlikely that experience would alter ratings, it is possible that more experienced learners or other allied health professionals may have different results.

## Conclusions

The findings of this study suggest that medical students’ ratings of physicians are altered by physical characteristics. It is significant since physical characteristics may be having an impact on the rating of physician preceptors. Further studies are required to confirm these findings and to try and determine their applicability in the real world.

## Additional file

Additional file 1: Copy of the questionnaire distributed to medical students. (DOCX 363 kb)
