# Supplementary material for: The effect of white coats and gender on medical students’ perceptions of physicians
Source: BMC Med Educ. 2017 May 26;17:93. doi: 10.1186/s12909-017-0932-1 (PMC5446716; doi:10.1186/s12909-017-0932-1)
Supplement: Additional file 1: — Copy of the questionnaire distributed to medical students. (DOCX 363 kb) [file 12909_2017_932_MOESM1_ESM.docx]

**Additional file 1**


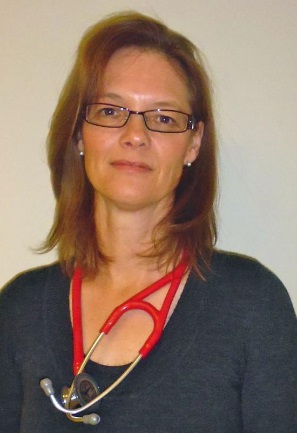

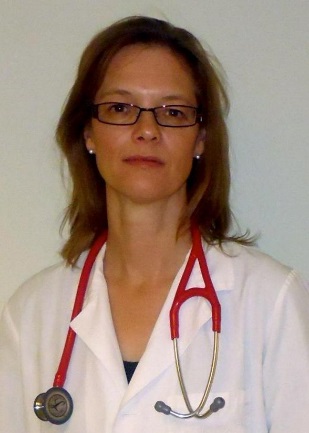
**(Note: Each group saw one of the two displayed pictures)**

A 79 year old gentlemen with moderate dementia was admitted to the hospital two weeks ago with *C.difficile colitis* from his nursing home. He has been treated with metronidazole for the last 2 weeks without success and he has also failed oral vancomycin. He continues to have 7-8 loose bowel movements per day. A family meeting is organized by the physician whereby she meets with the family to discuss the patient’s goals of care; it is decided that the patient will be moved from full resuscitation to comfort measures. At the time, this information is not directly communicated to the care team nor are they involved in the family meeting. Before the physician can write a note, she is called away to attend to another patient. Later that afternoon, the patient’s nurse is concerned because his respiratory status has worsened. The nurse is unable to find the doctor. Unaware of the previous meeting, the nurse is very concerned and calls a code due to respiratory failure whereby the patient is intubated.

Attending Physician

Based on the previous scenario please answer the following questions.

1. **How appropriately did the physician manage this situation?**

| 1  Not at all appropriately | 2  Somewhat  appropriately | 3  Neutral | 4  Appropriately | 5  Extremely appropriately |
| --- | --- | --- | --- | --- |

1. **Rate the physician’s competence.**

| 1  Not competent | 2  Somewhat  competent | 3  Neutral | 4  Competent | 5  Extremely competent |
| --- | --- | --- | --- | --- |

1. **Rate the physician’s trustworthiness.**

| 1  Not trustworthy | 2  Somewhat trustworthy | 3  Neutral | 4  Trustworthy | 5  Extremely trustworthy |
| --- | --- | --- | --- | --- |

1. **Rate the physician’s professionalism.**

| 1  Unacceptable | 2  Poor | 3  Satisfactory | 4  Good | 5  Excellent |
| --- | --- | --- | --- | --- |

1. **Has the physician committed a medical error?**

| 1  Definitely not | 2 | 3  Neutral | 4 | 5  Definitely has |
| --- | --- | --- | --- | --- |


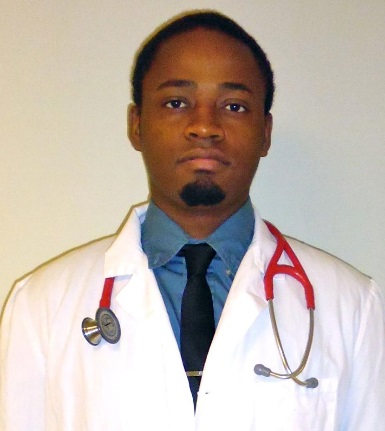

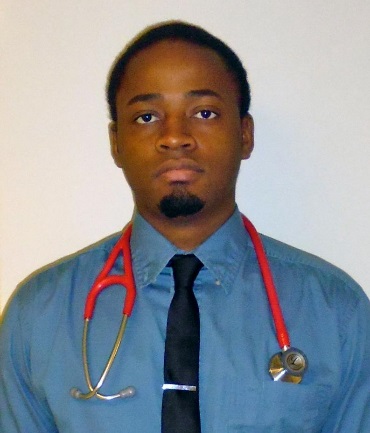


A 58 year old woman has been experiencing some heat intolerance and increased bowel movements over the past few months. On exam, the physician notes the patient has grade 3 brisk reflexes. Her labs indicate a suppressed level of thyroid-stimulating hormone (TSH). As per guidelines, she undergoes a technetium uptake scan, which shows a few areas of increased uptake in the thyroid. Given the symptoms and imaging, the patient and physician decide that she likely has a multinodular goiter and that she should see an Endocrinologist. After the patient leaves the clinic, the doctor fills out a referral and faxes it to the central triage system. Two weeks later, the patient calls the practice since she has not heard about a referral and is having escalating symptoms. The doctor realizes that the original fax that was sent did not go through.

Family Physician

Based on the previous scenario please answer the following questions.

1. **How appropriately did the physician manage this situation?**

| 1  Not at all appropriately | 2  Somewhat  appropriately | 3  Neutral | 4  Appropriately | 5  Extremely appropriately |
| --- | --- | --- | --- | --- |

1. **Rate the physician’s competence.**

| 1  Not competent | 2  Somewhat  competent | 3  Neutral | 4  Competent | 5  Extremely competent |
| --- | --- | --- | --- | --- |

1. **Rate the physician’s trustworthiness.**

| 1  Not trustworthy | 2  Somewhat trustworthy | 3  Neutral | 4  Trustworthy | 5  Extremely trustworthy |
| --- | --- | --- | --- | --- |

1. **Rate the physician’s professionalism.**

| 1  Unacceptable | 2  Poor | 3  Satisfactory | 4  Good | 5  Excellent |
| --- | --- | --- | --- | --- |

1. **Has the physician committed a medical error?**

| 1  Definitely not | 2 | 3  Neutral | 4 | 5  Definitely has |
| --- | --- | --- | --- | --- |


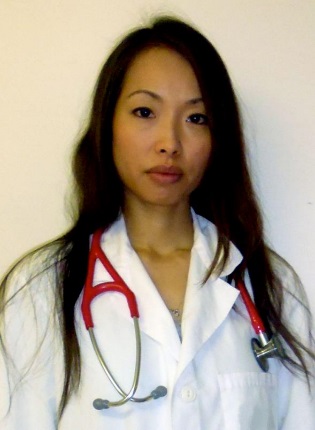

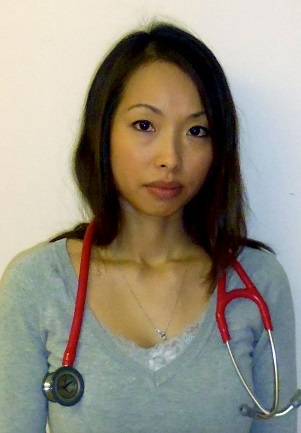
A 30 year old male comes to the emergency with generalized fatigue and severe muscle weakness which began in his lower limbs. The physician immediately orders standard bloodwork and an ECG. The patient’s labs demonstrate severe hyperkalemia (6.7 mEq/L) and an acute kidney injury with a creatinine of 750 micromol/L. His ECG reveals peaked T-waves. The physician deems this an emergency and obtains informed consent from the patient to insert a central line to perform hemodialysis. The nurse gathers an US machine, local anaesthetic and a general procedure tray as the doctor dresses in sterile protective gear. The physician uses the US to landmark the internal jugular vein and then proceeds with the process of inserting a hemodialysis catheter under ultrasound guidance. About halfway through the procedure, the physician thinks she may have accidentally touched the catheter to a non-sterile surface but decides to proceed with the current catheter since she does not want to delay therapy and thinks that the contamination is likely low risk. After the catheter insertion, the patient undergoes hemodialysis but twenty-four hours later, he develops a high-grade fever and redness near the central line insertion, likely due to a central

Emergency Physician

line-associated infection.

Based on the previous scenario please answer the following questions.

1. **How appropriately did the physician manage this situation?**

| 1  Not at all appropriately | 2  Somewhat  appropriately | 3  Neutral | 4  Appropriately | 5  Extremely appropriately |
| --- | --- | --- | --- | --- |

1. **Rate the physician’s competence.**

| 1  Not competent | 2  Somewhat  competent | 3  Neutral | 4  Competent | 5  Extremely competent |
| --- | --- | --- | --- | --- |

1. **Rate the physician’s trustworthiness.**

| 1  Not trustworthy | 2  Somewhat trustworthy | 3  Neutral | 4  Trustworthy | 5  Extremely trustworthy |
| --- | --- | --- | --- | --- |

1. **Rate the physician’s professionalism.**

| 1  Unacceptable | 2  Poor | 3  Satisfactory | 4  Good | 5  Excellent |
| --- | --- | --- | --- | --- |

1. **Has the physician committed a medical error?**

| 1  Definitely not | 2 | 3  Neutral | 4 | 5  Definitely has |
| --- | --- | --- | --- | --- |


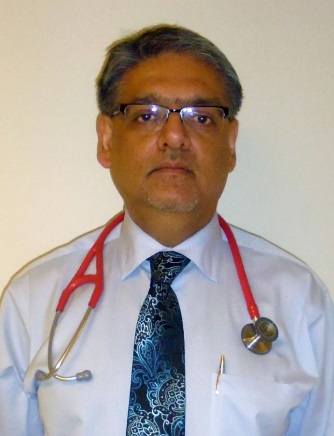

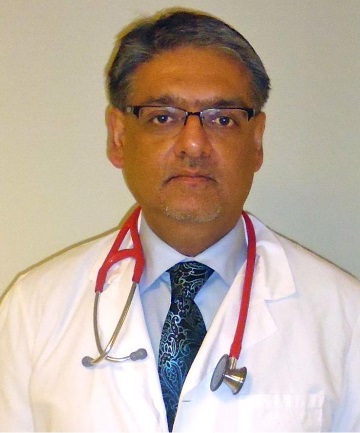
An 83 year old woman presents to the physician with general pain, a fever and a vesicles along her torso. The doctor sees the patient and diagnoses her with acute shingles and prescribes her 200 mg of acyclovir every 4 hours. Four days later, the physician is filing paperwork and realizes that he had neglected to check the patient’s kidney function and had neglected to account for her age prior to prescribing acyclovir. The doctor realizes that the patient is taking too high dose of acyclovir which could cause harm. The physician immediately phones the patient to check how she is doing; fortunately, the patient is experiencing no side effects. The physician titrates down the dose of acyclovir and orders appropriate follow-up testing which is normal.

Based on the previous scenario please answer the following questions.

Urgent Care Physician

1. **How appropriately did the physician manage this situation?**

| 1  Not at all appropriately | 2  Somewhat  appropriately | 3  Neutral | 4  Appropriately | 5  Extremely appropriately |
| --- | --- | --- | --- | --- |

1. **Rate the physician’s competence.**

| 1  Not competent | 2  Somewhat  competent | 3  Neutral | 4  Competent | 5  Extremely competent |
| --- | --- | --- | --- | --- |

1. **Rate the physician’s trustworthiness.**

| 1  Not trustworthy | 2  Somewhat trustworthy | 3  Neutral | 4  Trustworthy | 5  Extremely trustworthy |
| --- | --- | --- | --- | --- |

1. **Rate the physician’s professionalism.**

| 1  Unacceptable | 2  Poor | 3  Satisfactory | 4  Good | 5  Excellent |
| --- | --- | --- | --- | --- |

1. **Has the physician committed a medical error?**

| 1  Definitely not | 2 | 3  Neutral | 4 | 5  Definitely has |
| --- | --- | --- | --- | --- |
